# Cerebrospinal Fluid–Basic Concepts Review

**DOI:** 10.3390/biomedicines11051461

**Published:** 2023-05-17

**Authors:** Natalia Czarniak, Joanna Kamińska, Joanna Matowicka-Karna, Olga Martyna Koper-Lenkiewicz

**Affiliations:** Department of Clinical Laboratory Diagnostics, Medical University of Bialystok, 15-269 Bialystok, Poland; 37062@student.umb.edu.pl (N.C.); joanna.matowicka-karna@umb.edu.pl (J.M.-K.)

**Keywords:** cerebrospinal fluid, cerebrospinal fluid biomarkers, cerebrospinal fluid examination, cerebrospinal fluid storage, cerebrospinal fluid transport conditions, the blood–brain barrier, the blood-cerebrospinal fluid barrier

## Abstract

Cerebrospinal fluid plays a crucial role in protecting the central nervous system (CNS) by providing mechanical support, acting as a shock absorber, and transporting nutrients and waste products. It is produced in the ventricles of the brain and circulates through the brain and spinal cord in a continuous flow. In the current review, we presented basic concepts related to cerebrospinal fluid history, cerebrospinal fluid production, circulation, and its main components, the role of the blood–brain barrier and the blood–cerebrospinal fluid barrier in the maintenance of cerebrospinal fluid homeostasis, and the utility of Albumin Quotient (Q_Alb_) evaluation in the diagnosis of CNS diseases. We also discussed the collection of cerebrospinal fluid (type, number of tubes, and volume), time of transport to the laboratory, and storage conditions. Finally, we briefly presented the role of cerebrospinal fluid examination in CNS disease diagnosis of various etiologies and highlighted that research on identifying cerebrospinal fluid biomarkers indicating disease presence or severity, evaluating treatment effectiveness, and enabling understanding of pathogenesis and disease mechanisms is of great importance. Thus, in our opinion, research on cerebrospinal fluid is still necessary for both the improvement of CNS disease management and the discovery of new treatment options.

## 1. The History of Cerebrospinal Fluid

In the literature, the first mentions of cerebrospinal fluid appear as early as the 16th century B.C. in the Edwin Smith Papyrus, which contained 48 case reports related to head, spinal cord, and peripheral nerve injuries, each of which included a detailed description of examination, diagnosis, and treatment [1,2,3]. Later, in the 5th century B.C., Hippocrates’ notes on cerebrospinal fluid appeared, including the first description of the choroid plexuses of the lateral ventricles of the brain. Claudius Galen in the 2nd century C.E. presented the theory of three forms of pneuma: pneuma zoticon (vital spirit), pneuma physicon (natural spirit), and pneuma psychic (animal spirit). This theory held up for over a thousand years. Galen believed that pneuma entered the body through respiration. This ancient physician also described “vapours humor in the ventricles that provide energy to the entire body” [1,4,5,6].

In ancient times and the age of the Renaissance, autopsies were not widely practiced. If they were performed, they were always by decapitation, which precluded the study of cerebrospinal fluid. Progress in discovering the functions of the cerebrospinal fluid began in the age of the Renaissance when, in the year 1490, Leonardo da Vinci presented a wax cast of the ventricular system of the brain, with a high probability that it was based on Galen’s descriptions. However, it was Andreas Vesalius, the author of De humani corporis fabrica, who, in the 16th century, accurately illustrated the ventricles of the brain and noted that they were filled with aqueous humor, not gas. This discovery disproved Galen’s theory of three forms of pneuma [5].

In the years 1741–1744, Emanuel Swedenborg presented a detailed description of cerebrospinal fluid, which was published in 1887. Swedenborg described cerebrospinal fluid as “spirituous lymph” secreted from the atrium of the fourth ventricle into the medulla oblongata and spinal cord [1,4,5]. However, the breakthrough was made in 1842, when Francois Magendie defined cerebrospinal fluid as physiological fluid in the human body and named it “liquide cerebrospinal”. Magendie also accurately described the direction of cerebrospinal fluid flow, specifically the exit of cerebrospinal fluid from the fourth ventricle to the outside of the brain [1,4,6].

A few years later, in 1891, the German neurologist Heinrich Quincke described the method of lumbar puncture, enabling the safe collection of cerebrospinal fluid. Quincke performed lumbar puncture for the first time in children with increased levels of cerebrospinal fluid pressure, one of whom suffered from tuberculous meningitis [1,4,7]. Additionally, Quincke was the first to study the composition of cerebrospinal fluid in detail. Using the Kjdeldahl method, he measured the concentration of total protein, determined the number of cells, and detected the presence of bacteria in cerebrospinal fluid [1,5,7].

As one of the first, William Mestrezat collected data and presented the results of research on cerebrospinal fluid in many neurological diseases. His work in this field was considered a benchmark for many years [4,8]. The first attempt to measure the pressure of cerebrospinal fluid was carried out by the German neurologist Hans Queckensted. For this, he made use of a lumbar puncture needle that was connected to a U-shaped manometer. The cerebrospinal fluid, flowing through the manometer, was stopped by the back pressure, which was equal to the pressure of the cerebrospinal fluid [1,6]. Based on all of these discoveries, in 1925, Harvey Cushing recognized cerebrospinal fluid as the third circulatory system, alongside the vascular and lymphatic [4,5,9].

## 2. What Is Cerebrospinal Fluid?

Cerebrospinal fluid is a clear, colorless liquid produced primarily in the choroid plexus of the ventricular system but is also by the interstitial space of the brain and the subarachnoid space [10,11,12,13]. Cerebrospinal fluid fills the ventricles of the brain and the subarachnoid space, and its secretion by the cells of the choroid plexus is a two-stage process. In the first step, the plasma is passively filtered through the capillary endothelium into the interstitial space of the choroid, this step occurring due to the osmotic pressure gradient between these structures. In the second stage, plasma ultrafiltrate is actively transported via the epithelial cell membrane of the choroid plexus with the participation of transport proteins [10,14,15]. The choroid plexuses are branched structures composed of a large number of blood vessels [16]. Epithelial cells of the choroid plexus are involved in the formation of the blood–cerebrospinal fluid barrier (BCB), due to the presence of intercellular connections, so-called tight junctions. The presence of tight junctions prevents the free movement of the cerebrospinal fluid [13,14,17].

## 3. Cerebrospinal Fluid Production and Circulation

Cerebrospinal fluid is produced by passive ultrafiltration of the fluid through the capillaries of the choroid plexus and by active transport of ions by the endothelial cells of the choroid plexus [18,19]. An important role in the production of cerebrospinal fluid is carried out by carbonic anhydrase and membrane proteins transported ions. Carbonic anhydrase is an enzyme that converts carbon dioxide (CO_2_) and water (H_2_O) to carbonic acid (H_2_CO_3_) in a reversible manner. Then, H_2_CO_3_ dissociates into hydrogen ions (H^+^) and bicarbonate ions (HCO_3_^−^) [15,16,18]. In the apical (luminal) part of the membrane of endothelial cells of the choroid plexus, H^+^ ions are transported from the inside of the cell to the cerebrospinal fluid by the sodium-hydrogen exchanger (NHE) transporter. The NHE transporter as an antiporter exchanges one H^+^ ion for one sodium (Na^+^) ion [20]. The transport of Na^+^ ions from inside the cell to the cerebrospinal fluid is carried out by the sodium–potassium pump (ATPase Na^+^/K^+^), which exchanges three Na^+^ ions for two K^+^ ions [19,20,21,22]. The Na^+^/K^+^ ATPase pump also provides energy for other active ion exchanges needed to produce cerebrospinal fluid [21]. The transfer of K^+^ ions from the inside of the cell to the cerebrospinal fluid takes place with the participation of the inward-rectifier potassium channel (Kir), which is gated by changes in the membrane potential of the cell [16]. HCO_3_^−^ ions are transported to epithelial cells from the cerebrospinal fluid by the sodium bicarbonate co-transporter (NBCn1) [23]. NBCn1 expression was also demonstrated in the basolateral part of the membrane of endothelial cells of the choroid plexus [23]. In the basolateral membrane of the choroid plexus, endothelial cells and HCO_3_^−^ ions are transported from the epithelial cells into the blood by the anion exchange protein 2 (AE2) ion carrier protein (Cl^−^/HCO_3_^−^ exchanger) [21,23]. Chloride (Cl^−^) ions that have entered choroid plexus epithelial cells via AE2 are then transported to the cerebrospinal fluid via the Na^+^/K^+^/2Cl^−^ (sodium potassium chloride co-transporter, NKCC1), K^+^/Cl^−^ co-transporter (potassium-chloride co-transporter, KCC4), and by inward-rectifying chloride channel (Clir) and volume-regulated anion channel (VRAC), which are gated by potential cell membrane changes [16,21,22,23]. The potassium chloride cotransporter 1 (KCC1) is responsible for the outflow of K^+^ and Cl^−^ ions from the epithelial cells of the choroid plexus into the blood [24]. In the basolateral membrane of the choroid plexus endothelial cells, there are transport proteins NBCn1 and sodium bicarbonate cotransporter 2 (NBCn2), which enable the transport of Na^+^ and HCO_3_^−^ ions into the cell [16,24]. It should be noted that NBCn2 refers to humans, while this transport protein may be referred to as NCBE in other species [24,25].

**Figure 1:**
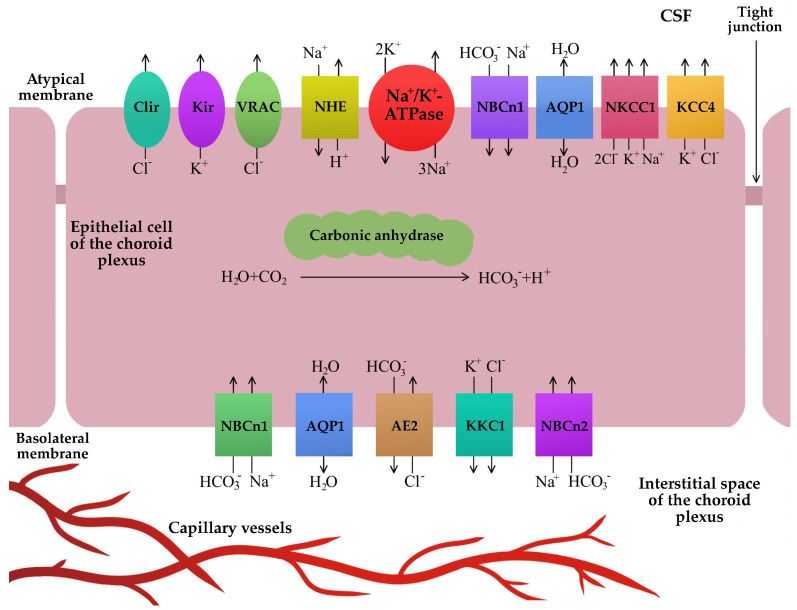
Cerebrospinal fluid production. Its production is based on the active exchange of ions and H_2_O between the interstitial space of the choroid plexus and the cerebrospinal fluid. Carbonic anhydrase catalyzes the conversion of H_2_O and CO_2_ to H^+^ and HCO_3_^−^ ions. Ion carrier proteins transport Na^+^, Cl^−^, and HCO_3_^−^ ions from the extracellular fluid through the basolateral membrane into the choroid plexus epithelial cells and then, after intracellular circulation, through the apical membrane of the choroid plexus epithelial cells into the cerebrospinal fluid. H_2_O enters the choroid plexus epithelial cells mainly through AQP1 as a result of the osmotic pressure gradient. AE2—anion exchange protein 2; AQP1—aquaporin 1; Cl^−^—chloride ions; Clir—inward-rectifying chloride channel; CO_2_—carbon dioxide; CSF—cerebrospinal fluid; H^+^—hydrogen ions; H_2_O—hydrogen monoxide, water; HCO_3_^−^—bicarbonate ions; K^+^—potassium ions; KCC1—potassium chloride cotransporter 1; KCC4—potassium-chloride co-transporter 4; Kir—inward-rectifier potassium channel; Na^+^—sodium ions; ATPase Na^+^/K^+^—sodium-potassium pump; NBCn1—sodium bicarbonate co-transporter 1; NBCn2—sodium bicarbonate co-transporter 2; NHE—sodium-hydrogen exchanger; NKCC1—sodium potassium chloride co-transporter 1; VRAC—volume-regulated anion channel.

The influx of ions into the cerebrospinal fluid results in the presence of an osmotic pressure gradient, enabling the transport of H_2_O by aquaporin 1 (AQP1). Aquaporin channels are responsible for regulating the volume of the extracellular space, the circulation of the cerebrospinal fluid, and the absorption of interstitial fluid [15,21,26]. They are present in both the basolateral and apical membranes of the endothelial cells of the choroid plexus [21]. Alternatively, H_2_O can be transported independently of the osmotic pressure gradient. It has been shown that about half of the cerebrospinal fluid is produced by the co-transport of water and ions through the sodium potassium chloride co-transporter (NKCC1) channels located in the apical membrane of the choroid plexus epithelial cells [24]. Figure 1 presents a schematic production of cerebrospinal fluid (Figure 1).

The cerebrospinal fluid flow consists of a combination of directed and pulsating flow from its production site to the place where it is reabsorbed [9,15,21]. Cerebrospinal fluid is mostly produced in the two lateral ventricles of the brain, from which it flows through the foramen of Monroe to the third ventricle and then through the aqueduct of Sylvius to the fourth ventricle. From the fourth ventricle, the cerebrospinal fluid flows through the foramen Magendi and the two lateral foramina of Luschka into the subarachnoid space of the brain and the spinal cord [9,10,14,15,22,26,27].

**Figure 2:**
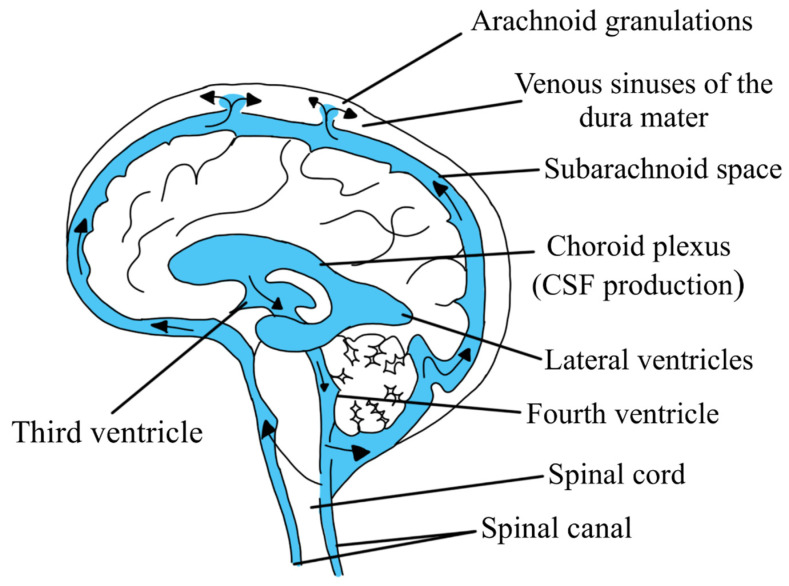
Cerebrospinal fluid flow. Cerebrospinal fluid is mainly produced in the lateral ventricles of the brain, while being produced to a smaller extent in the third and fourth ventricles. From the lateral ventricles of the brain, the cerebrospinal fluid flows through Monroe’s foramen into the third ventricle, and it flows from there through the aqueduct of Sylvius into the fourth ventricle, from where it flows through the Magendi’s foramen and two lateral foramina of Luschka into the subarachnoid space of the brain and the spinal cord. Cerebrospinal fluid is mainly absorbed through the arachnoid granulations into the dural venous sinuses and from there into the blood. Arrows shows direction of cerebrospinal fluid flow.

Reabsorption of cerebrospinal fluid is not as complex as its production process; it occurs through the arachnoid granulations into the venous sinuses of the dura mater and from there it enters the blood (Figure 2). In addition, part of the cerebrospinal fluid may be reabsorbed by the choroid plexus to flow into the cervical and thoracic lymphatic vessels [9,10,12,14,15]. Factors permitting the flow of cerebrospinal fluid are the forces generated by the pulsations of the heart and the movement of the lungs [10,14,15,28]. In adults, the total volume of cerebrospinal fluid is about 150 mL, while its production rate is about 20 mL/h, or 500 mL/day [14,15,16,26,27,28].

The choroid plexus controls cerebrospinal fluid secretion, which is innervated by the autonomic, cholinergic, adrenergic, serotonergic, and peptidergic nervous systems [15]. Stimulation of the sympathetic nervous system reduces the secretion of cerebrospinal fluid, while stimulation of the cholinergic system increases its production [15,26].

Additionally, the volume of cerebrospinal fluid can be regulated by using inhibitors/activators of the relevant membrane proteins of the choroid plexus epithelial cells. The above-mentioned regulation is closely related to the ATPase Na^+^/K^+^. Administration of corticosteroids may reduce the activity of the ATPase Na^+^/K^+^, consequently reducing the production of cerebrospinal fluid [29].

The main component of cerebrospinal fluid is water [27,30]. Therefore, the regulation of cerebrospinal fluid secretion is also dependent on the activity of carbonic anhydrase. Carbonic anhydrase inhibitors that reduce the production of cerebrospinal fluid are acetazolamide and furosemide, mainly used in the treatment of hydrocephalus [29,31]. Hormones regulating the body’s water homeostasis also affect cerebrospinal fluid volume [26]. Vasopressin (antidiuretic hormone, ADH) acts on the arteries of the choroid plexus. By constricting these vessels, vasopressin can reduce blood flow to the choroid plexus, decreasing the volume of circulating cerebrospinal fluid [29]. Such drugs as thiopental, midazolam, and etomidate also reduce cerebrospinal fluid secretion by reducing cerebral blood flow and cerebral oxygen metabolism. In turn, substances that stimulate cerebrospinal fluid production include ketamine and enflurane. Ketamine’s mechanism of action is to increase cerebral blood flow, while enflurane increases choroid plexus metabolism [29].

## 4. Cerebrospinal Fluid Components

**Figure 3:**
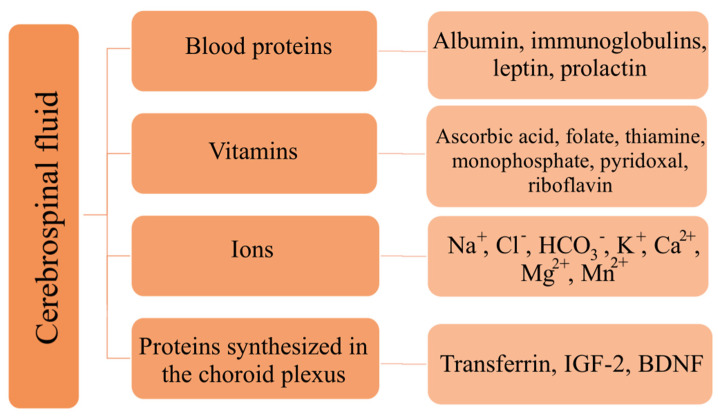
Examples of substances present in cerebrospinal fluid. BDNF—brain-derived neurotrophic factor; Ca^2+^—calcium ions; Cl^−^—chloride ions; HCO_3_^−^—bicarbonate ions; IGF-2—insulin-like growth factor 2; K^+^—potassium ions; Mg^2+^—magnesium ions; Mn^2+^—manganate ions; Na^+^—sodium ions.

The main component of cerebrospinal fluid is water (99%), with the remaining part (1%) made up of protein, glucose, ions, vitamins, and neurotransmitters. The composition of cerebrospinal fluid is very similar to that of plasma, except for the difference in protein and ion concentrations [14,27,30]. Table 1 presents a comparison of the composition of cerebrospinal fluid and plasma in adults (Table 1) [32]. Figure 3 presents examples of substances present in cerebrospinal fluid (Figure 3) [13].

## 5. The Role of Cerebrospinal Fluid

Cerebrospinal fluid as a liquid surrounding the brain on all sides primarily protects it against shocks and the risk of damage arising from contact with the skull. The average weight of an adult human brain is about 1500 g, but, due to the presence of the surrounding cerebrospinal fluid and buoyancy, this weight is reduced by 10–15 times [11,12,16,21]. In addition to protecting the brain, cerebrospinal fluid is also responsible for transporting substances necessary for the functioning of the CNS and eliminates waste products and toxic substances emanating from it. In addition, it affects the maintenance of CNS homeostasis by regulating the concentration of electrolytes and transporting neurotransmitters and hormones [12,14,16,21]. Cerebrospinal fluid is also of great importance in the diagnosis of CNS diseases of various etiologies. Through the cerebrospinal fluid, it is also possible to administer drugs to the CNS which ordinarily would not be able to be transported from the blood due to the presence of the blood–brain barrier (BBB) [21,33].

## 6. Blood–Brain Barrier (BBB)

The proper maintenance of CNS homeostasis is ensured by two barriers, the BBB and the blood–cerebrospinal fluid barrier (BCB). The BBB is an anatomical barrier responsible for separating circulating blood from the extracellular space of the brain. The main components of this barrier are endothelial cells of the brain capillaries, pericytes, and astrocytes [11,12,34,35,36,37,38].

The most important elements of the BBB are the endothelial cells lining the inner layer of the capillary wall of the brain. These cells are highly specialized, their intercellular spaces being covered with a large number of expanded connections [36,39,40]. The endothelial cells of the brain capillaries differ from other endothelial cells of our body primarily due to low pinocytic activity and many mitochondria [16,35,36,40,41].

Astrocytes are another building block of the BBB. They are mainly responsible for nutrition, neurotransmission, and excretion of metabolic products. Astrocytes are the link between the blood and the brain by connecting one end to capillaries of the brain and the other end to neurons. This neurovascular connection allows for the transmission of signals that regulate blood flow [40]. The ends of astrocytes resemble cap-like structures are called end feet and cover the walls of the capillaries [12,37,41]. The end feet of astrocytes contain a large number of AQP water channels and Kir4 channels, which play a key role in the movement of cerebrospinal fluid into the brain parenchyma [39,41]. Astrocytes can also secrete growth factors such as transforming growth factor-b (TGFb), basic fibroblast growth factor (bFGF), angiopoietin 1 (ANG 1), and glial-derived neurotrophic factor (GDNF) [40,41].

**Figure 4:**
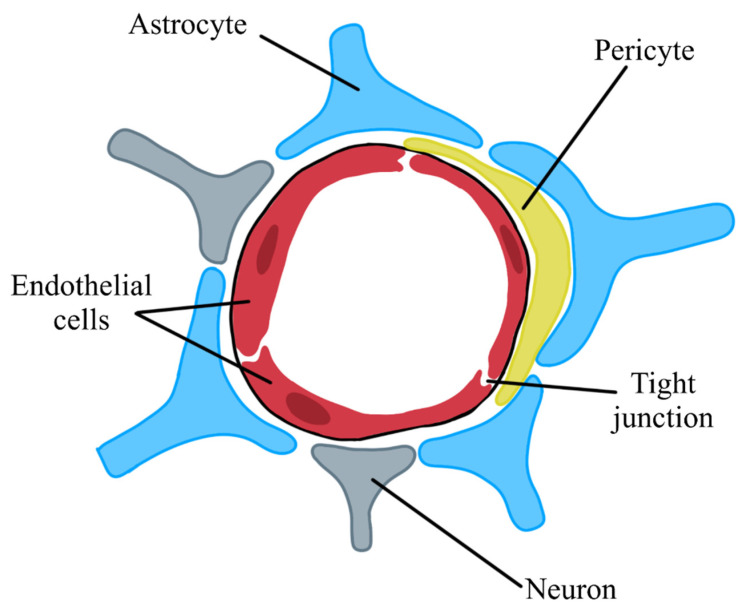
Structural components of the blood–brain barrier. Blue—astrocyte, yellow—pericyte, gray—neuron, red—endothelial cells.

The last element of the BBB are pericytes, found in the brain as regulators of barrier functions and mediators of neuritis. Pericytes are located near the blood vessels, which facilitate the regulation of the immune and inflammatory response [38,39,42]. Additionally, pericytes can phagocytose and can affect the diameter of the capillaries of endothelial cells and are also a source of angiopoietin. Therefore, they are responsible for maintaining the continuity of the BBB between endothelial cells in the brain [34,42]. Figure 4 presents the structural components of the BBB (Figure 4).

The BBB owes its functionality to the presence of specific tight junctions between epithelial cells—these are adherens junctions (AJs) and tight junctions (TJs). On the surfaces of the apical membranes of the endothelial cells of the capillaries of the brain, there are TJs [43,44]. They consist of various subunits of transport proteins such as occludins, claudins, cadherins, and adhesion molecules JAM-A, -B, -C, and -D [40,41,43,44]. TJs have two main functions, the first is to prevent the mixing of membrane proteins between the apical and basolateral membranes, and the second is to control ion and solute transport [41]. The occludins are a family of transmembrane proteins with a molecular weight of approximately 60 kDa. The main function of occludins is to control the ion selectivity and permeability of the transcellular pathway between cells [41,45] The claudins are a family of over 24 proteins with a molecular weight of 20–27 kDa. They contain two extracellular loops and four transmembrane domains. Their proper interaction between adjacent endothelial cells is essential to maintain BBB tightness by selectively regulating ion transport [40,41,43,45,46]. Claudins and occludins, via the proteins of the zonula occludens complex (ZO-1, -2, -3), connect to elements of the membrane cytoskeleton [36,37,43]. JAM proteins (-A, -B, -C, and-D) also influence the functioning of the BBB. They are immunoglobulins with a molecular weight of 40 kDa, their function not being fully understood. It is supposed that they play a role in cell adhesion and leukocyte migration [36,37,41]. The ZO-1, -2, and -3 proteins belong to the family of membrane guanylate kinase homologues. All three proteins form a complex linked to the C-terminus of occludin and the other end to cingulin. This family has a PDZ domain, SH3, and a guanylate kinase homology domain [43,45,46,47]. Their function is to maintain the integrity of the BBB by linking the intracellular domains of claudins, occludins, and cell adhesion proteins to the actin skeleton of the endothelial cell [40,41,45].

**Figure 5:**
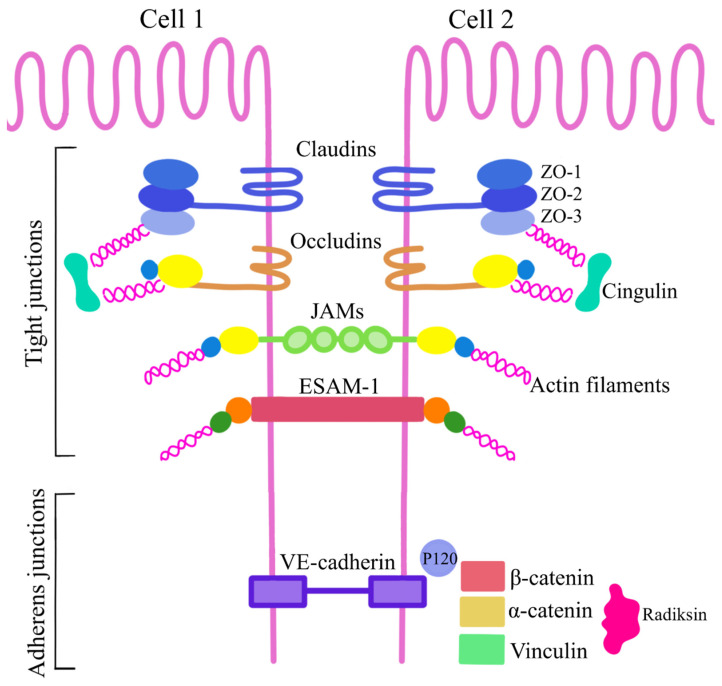
Illustration of the junction between two brain capillary endothelial cells that make up the blood–brain barrier. Tight junctions (TJs) consist of various subunits of transport proteins such as occludins, claudins, cadherins, and JAM adhesion molecules. Adherens junctions (AJs) are responsible for the initiation and stabilization of intercellular adhesions and regulate the actin cytoskeleton of endothelial cells of brain capillaries. ESAM—endothelial cell-selective adhesion molecule; JAM-A—junctional adhesion molecule; PECAM-1—platelet–endothelial cell adhesion molecule-1; VE-cadherin—vascular endothelial-cadherin; ZO—zonula occludens.

The second type of connection, thanks to which the BBB can properly fulfill its function, is linked to AJs. They are located between the basement membrane and endothelial cells. They consist of cadherins, which are calcium (Ca^2+^)-dependent transmembrane glycoproteins. Cadherins bind to beta-catenin and plakoglobin (P120), which then, via beta-catenin, alpha-catenin, vinculin, and radixin, stabilize AJs by binding to the cell cytoskeleton [41,45,47]. AJs are responsible for the initiation and stabilization of intercellular adhesions and regulate the actin cytoskeleton [41,45]. Figure 5 presents a diagram of the connections between the endothelial cells of the brain capillaries that are part of the BBB (Figure 5).

Two proteins, the neuron-specific enolase (NSE) and the S100 protein, are used in routine laboratory practice to assess the integrity of the BBB. NSE exists in various dimeric isoforms and consists of α, β, and γ subunits. NSE γ is a glycolytic enzyme found in neurons and endocrine cells. An elevated concentration of NSE in the serum and cerebrospinal fluid is indicative of damage to the nervous tissue, which may result from stroke, epilepsy, hypoxia, or cancer [48,49,50,51,52]. The S100 protein belongs to a family of Ca^2+^ binding proteins. The 100A1 and 100B protein genes are expressed in CNS cells, mainly in astroglial cells, as well as in melanoma cells and other tissues. S100 protein concentration increases in cerebrospinal fluid and blood as a result of stroke or trauma that leads to brain damage [51,53,54,55].

## 7. Blood–Cerebrospinal Fluid Barrier (BCB)

**Figure 6:**
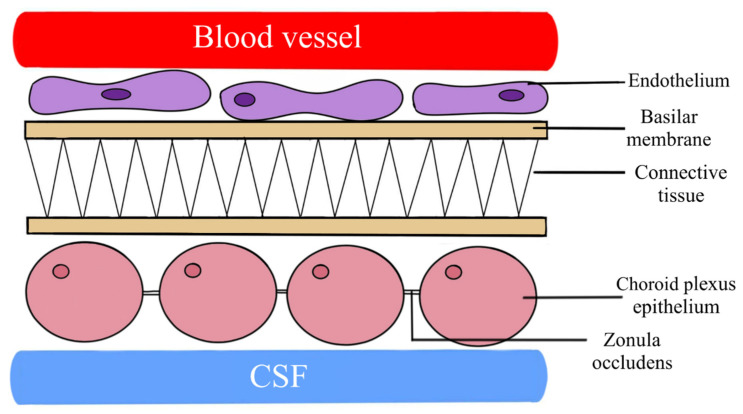
A schematic diagram presenting the blood–cerebrospinal fluid barrier (BCB). Functionally, BCB is a set of mechanisms that allow proteins to flow from the blood into the cerebrospinal fluid. BCB is formed by epithelial cells of the choroid plexus of the four ventricles of the brain and epithelial subarachnoid structures directed in the intracranial areas and the spine to the cerebrospinal fluid space.

BCB, unlike BBB, is not assigned to a precise location, being functionally related to the choroid plexus. In functional terms, BCB is a series of mechanisms responsible for the diffusion of proteins from the blood to the cerebrospinal fluid. It is formed by epithelial cells of the choroid plexus of the four ventricles of the brain and subarachnoid epithelial structures directed into the cerebrospinal fluid space in the intracranial areas and the spine [12,28,36,38]. Figure 6 presents a morphological diagram of the BCB (Figure 6). The increase in BCB permeability occurs as a result of the slowing of the flow rate of the cerebrospinal fluid. This results in an increase in total protein concentration, including albumin, in the cerebrospinal fluid and an increase in the albumin quotient (Q_Alb_) [12,15,56,57,58]. Neurological diseases leading to the slowing of the flow of cerebrospinal fluid include, among others: purulent bacterial meningitis, CNS leukemia, Froin syndrome, meningeal carcinoma, Guillain–Barré syndrome, and multiple sclerosis [56].

## 8. Albumin Quotient (Q_Alb_)

The albumin quotient (Q_Alb_) is used to assess BCB functionality. It expresses the ratio of the concentration of albumin in the cerebrospinal fluid to the concentration of albumin in the serum [12,39,59,60,61]. Thus, the calculation of Q_Alb_ requires that the patient’s blood be drawn into a tube without anticoagulant to obtain serum and cerebrospinal fluid [62]. Albumin in the cerebrospinal fluid is derived only by simple diffusion from the blood and is not used by CNS cells [60,63]. With increased diffusion of albumin from the blood into the cerebrospinal fluid, the value of Q_Alb_ increases, indicating BCB dysfunction. There is no lower range for Q_Alb_. The Q_Alb_ upper range is age dependent and should be calculated using the formula: Q_Alb_ = (age in years/15) + 4. However, the formula is only used for people aged 15–60. Physiologically, newborns have an elevated concentration of albumin in the cerebrospinal fluid, and thus an increased value of Q_Alb_. Then, with every passing month, this value decreases and is at its lowest in the 4th month of life. From the 4th month, the value of Q_Alb_ increases, and, in children aged 15 years, it is at the level of 5.00 × 10^−3^ [62,63]. In children up to 15 years of age, the upper range of Q_Alb_ is determined using a graph showing the relationship between Q_Alb_ and age. For people over 60 years of age, the upper range is 8.00 × 10^−3^ [58,62,63].

The units used to express albumin concentration in serum (g/dL) and cerebrospinal fluid (mg/dL) are different because the range of concentrations in these two materials differs. Units must be standardized when calculating Q_Alb_, as it would be inappropriate to divide different units together. As a result of division, a fraction is obtained, which is inconvenient in everyday practice. Therefore, Q_Alb_ is usually given as an integer number multiplied by 10^−3^ [63].

A mild increase in Q_Alb_ (8–25 × 10^−3^) can be observed in diseases such as immune-mediated polyneuropathy and viral meningitis. A significant increase in Q_Alb_ > 25 × 10^−3^ may indicate purulent meningitis, acute neuroborreliosis, immune-mediated myelitis, or Guillain–Barré syndrome [63,64].

## 9. The Collection and Storage of Cerebrospinal Fluid

**Figure 7:**
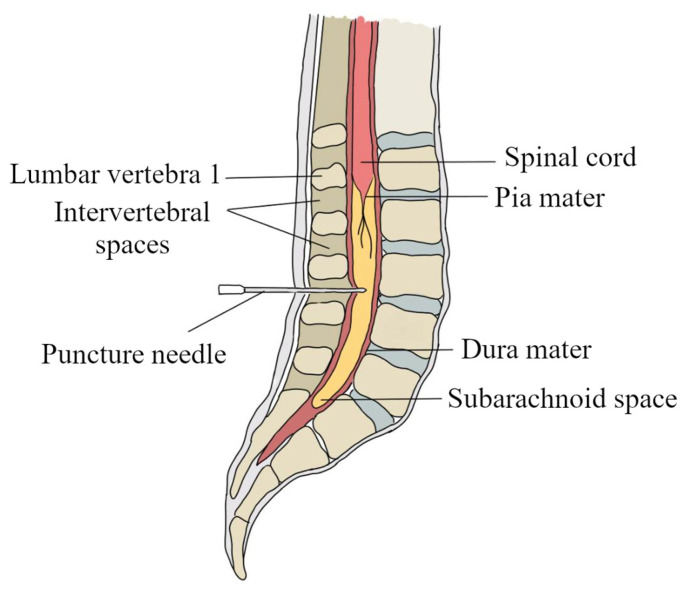
Diagram showing the site of cerebrospinal fluid collection by lumbar puncture. To perform a lumbar puncture, a puncture needle should be placed between the 3rd and 4th or 4th and 5th lumbar vertebrae. The needle is then inserted into the subarachnoid space where the cerebrospinal fluid is located.

**Figure 8:**
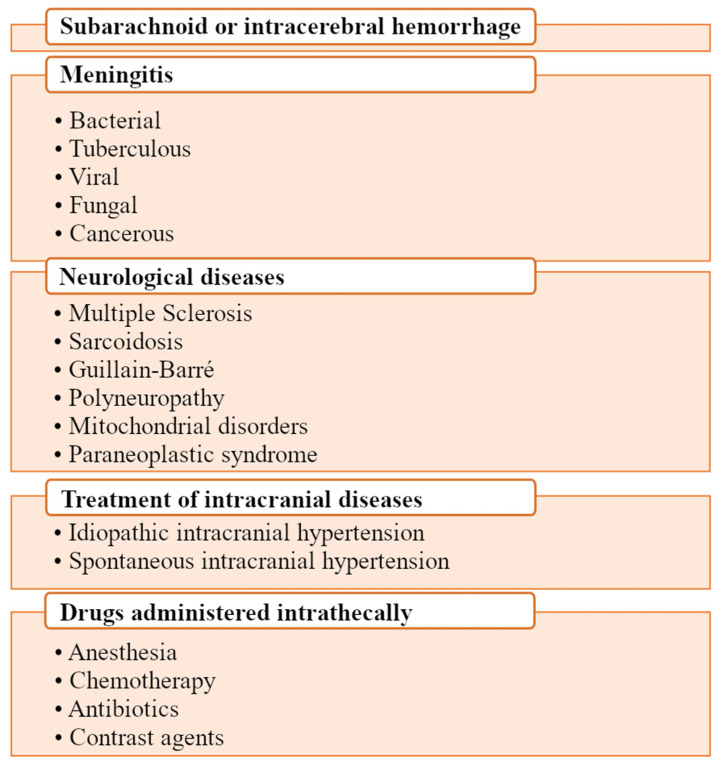
Indications for cerebrospinal fluid collection. The main purpose of collecting cerebrospinal fluid is the diagnosis of CNS diseases. In addition, drugs that do not penetrate from the blood into the CNS can also be administered via a lumbar puncture.

The collection of cerebrospinal fluid is an invasive procedure and may be associated with post-puncture syndrome [65,66]. The procedure for collecting cerebrospinal fluid is performed by lumbar puncture, sub occipital puncture, or collection directly from the lateral ventricles of the brain through external ventricular drainage [67,68]. The most common way to collect cerebrospinal fluid is through lumbar puncture, which involves inserting a puncture needle into the subarachnoid space. The patient from whom the fluid is collected should be positioned appropriately, i.e., on the side with legs bent at the knee joint and arms bent at the elbow joint, drawn to the chest. Lumbar puncture in an adult should be made between the 3rd and 4th or 4th and 5th intervertebral space of the lumbar spine (Figure 7) [65,69,70,71]. During the collection of cerebrospinal fluid, the puncture needle penetrates the following structures in the following order: skin, subcutaneous tissue, supraspinatus ligament, interspinous ligament, flat ligament, epidural space containing the inner vertebral venous plexus, dura mater, arachnoid up to the subarachnoid space [65,66,71]. It is recommended that a physician use a manometer attached to a spinal needle to measure the “opening” pressure of cerebrospinal fluid. The normal range for cerebrospinal fluid pressures in an adult in the lateral recumbent position is between 50–180 mm Hg, with slightly higher pressures obtained from individuals who are sitting. If the pressure falls within the normal range, it is safe to collect approximately 15% (about 20 mL) of the total volume of cerebrospinal fluid. Following a cerebrospinal fluid puncture, the physician can check the “closing” pressure, which should be 10–30 mm Hg lower than the “opening” pressure [72]. Examples of indications for a lumbar puncture are shown in Figure 8 [65,73].

Contraindications associated with the lumbar puncture procedure include local skin infections, abnormalities of the skin or spine in the puncture area, sepsis, and increased intracranial pressure. Coagulation disorders and anticoagulant treatment also disqualify the patient from undergoing this procedure. Lumbar puncture is an invasive procedure and carries the risk of complications in the form of brain herniation. One of the most common complications of puncture is a headache caused by a decrease in pressure in the subarachnoid space [27,65,70,74]. Very large reductions in intracranial cerebrospinal fluid volume can also be related to post-puncture headache, but, sometimes, headaches can occur with relatively little alteration of cerebrospinal fluid volume [75]. Therefore, atraumatic needles are believed to lower the incidence of post-dural-puncture headache by minimizing the loss of cerebrospinal fluid after lumbar puncture [76]. Moreover, patients who experience headache prior to puncture are at higher risk of developing post-lumbar puncture headache. Additionally, Kuntz et al. [77] suggest that younger female patients with a lower body mass index are at the highest risk of developing post-puncture headaches.

Other complications may include local bruising, nausea, bleeding, and discomfort associated with the procedure. Extremely dangerous is iatrogenic meningitis and paresis of the limbs resulting from the formation of a subdural hematoma. The lumbar puncture procedure may damage the blood vessels with the puncture needle. Contamination of the cerebrospinal fluid with blood is referred to as traumatic puncture (or traumatic tap) and makes it difficult to interpret the result of the laboratory examination of the cerebrospinal fluid [27,66,69,74]. The presence of red blood cells in a traumatic tap situation can lead to falsely elevated protein concentration and white blood cell count in the cerebrospinal fluid [78]. Therefore, it is recommended that the cerebrospinal fluid should be collected in a minimum of three tubes [70], as traumatic tap can result in the greatest amount of red blood cells in the cerebrospinal fluid collected in the first tube. Thus, a significant difference can be observed by visually examining or comparing the red blood cell count between the first and third tubes, indicating a traumatic tap with the highest concentration of red blood cells in the first tube. Conversely, a subarachnoid hemorrhage may show a uniform distribution of red blood cells across all collection tubes. Furthermore, the centrifugation of the cerebrospinal fluid can help distinguish between the two conditions. A colorless supernatant indicates a traumatic tap, whereas a yellow-colored supernatant (xanthochromic) suggests a hemorrhage, as it takes around 1–2 h for red blood cells to lyse in cerebrospinal fluid [72].

The collection of cerebrospinal fluid by suboccipital puncture is dangerous due to the collection site being close to the medulla oblongata; therefore, it is not routinely performed. Collection of cerebrospinal fluid from the lateral ventricles is performed in infants with unfused fontanelles or intraoperatively in adults through intraventricular drains. If the cerebrospinal fluid was collected by a method other than a lumbar puncture, this information should be included in the referral and the laboratory examination result [36,51,79].

Cerebrospinal fluid should not be aspirated as hypertension may increase the risk of hernia [70]. To date, standards for the number and volume of test tubes, as well as the time and conditions for transporting cerebrospinal fluid to the laboratory have not been unified [70,73,80]. Wright et al. [70] proposed that the amount of cerebrospinal fluid needed to determine glucose concentration is about 0.5 mL—while, for the study of oligoclonal bands, about 0.1 mL—and about 20 mL is needed for microbiological tests.

In turn, Gastaldi et al. [73] suggested that the amount of cerebrospinal fluid needed for laboratory testing should be 4–5 mL, while Brunstein et al. and Lygirou et al. [69,80] suggested an amount of 1–2 mL. In the laboratory of the authors of the current publication, for the collection of the cerebrospinal fluid into three tubes, 1–2 mL each is recommended.

For cerebrospinal fluid collection polypropylene tubes with low protein binding capacity should be used. The use of laboratory plastic or glass may cause protein adhesion, which affects laboratory examination results [81,82]. Sterile tubes made of siliconized glass are also permitted to be used, but the disadvantage of this material is the increased adhesion of monocytes [73]. In the laboratory of the authors of the current publication, it is recommended that biochemical and immunological tests should be performed from the first tube, microbiological tests from the second tube, and the third tube should be used for cytological tests. It is also important to collect blood in a tube without an anticoagulant to obtain serum to assess albumin and glucose concentration to calculate Q_Alb_ and Glucose Index, respectively [69,73,81,82]. The cerebrospinal fluid should be delivered to the laboratory within 30 min, but no later than 2 h, after collection [81,82]. In the laboratory of the authors of the current publication, it is recommended that the cerebrospinal fluid for microbiological testing should be delivered at 37 °C. According to Deisenhammer et al. [32] if it is necessary to store the cerebrospinal fluid for later analysis, the sample can be stored short-term at 4–8 °C or long-term at −20 °C [32]. The authors of the current publication store cerebrospinal fluid for scientific research at −75 °C. If rapid transport is not possible, the cerebrospinal fluid should be stored in appropriate conditions that ensure the stability of the parameters tested (Table 2) [72].

## 10. Meningeal Lymphatic Vessels and Neurological Diseases

Although Paolo Mascagni was the first to describe the cerebral vascular lymphatic system at the end of the 18th century, his discovery was not widely accepted at the time [83]. Thus, for a long time, the CNS has been considered an immune-privileged site, owing to the absence of parenchymal lymphatic vessels required for the transportation of antigens to the lymph nodes. Further works suggesting the presence of lymphatic vessels in the brain appeared in the 21st century [84,85]. However, it was only in 2015 that research was published that provided conclusive evidence for the existence of lymphatic vessels within the dura mater in rodents, primates, and humans [83,86,87]. These vessels expressed all molecular markers of endothelial lymphoid cells, such as prospero homeobox protein 1 (PROX1) transcription factor, vascular endothelial growth factor receptor 3 (VEGFR3), a lymphangiogenic tyrosine kinase receptor, chemokine (C-C motif) ligand 21 (CCL21), lymphatic vessel endothelial hyaluronan receptor 1 (LYVE1), and podoplanin [88]. The lymphatic vessel system, running alongside blood vessels and controlled by glial cells, has been termed the glymphatic system [86,87].

Further studies have shown that the meningeal lymphatic vessels play an important role in clearing macromolecules from the cerebrospinal fluid and interstitial fluid, transporting them to the deep cervical lymph nodes, and in transporting immune system cells from the CNS to these nodes [86,87,88,89,90,91]. In addition, research by Ahn et al. [89] showed that meningeal lymphatic vessels have specialized functions and structure, depending on their location inside the skull (i.e., in the dorsal or basal region). The dorsal meningeal lymphatic vessels are situated in the dural folds, particularly along the superior sagittal sinus and transverse sinus. These vessels possess small diameters and mostly exhibit discontinuous vascular structures. The basal meningeal lymphatic vessels, which follow the path of the petrosquamosal sinus and sigmoid sinus, display larger diameters and numerous protruding capillary branches with blunt ends. These branches are characterized by typical oak-leaf-shaped lymphatic endothelial cells and lymphatic valves that resemble those found in functional classic lymphatic vessels [89]. Ahn et al. [89] in a mouse model showed that the basal meningeal lymphatic vessels are more involved in macromolecular drainage and cerebrospinal fluid clearance compared to the dorsal ones. This is mainly due to their anatomical location near the subarachnoid space and their characteristic structure, including lymphatic capillaries with blunt-ended protrusions and a predominantly button-like junctional pattern, as well as lymphatic valves that resemble those found in pre-collectors. The authors also showed that basal meningeal lymphatic vessels are susceptible to changes associated with lymphedema, which is manifested by valvular dysfunction in older mice [89].

Studies conducted thus far suggest that reduced macromolecular drainage and impaired clearance of cerebrospinal fluid by meningeal lymphatic vessels may be the cause of the development of neuroinflammatory and neurodegenerative diseases [85,89,90,92,93]. Changes in the functioning of meningeal lymphatic vessels have been observed in subarachnoid hemorrhage, Parkinson’s disease, Alzheimer’s disease, vascular dementia, brain tumors, multiple sclerosis, traumatic brain injury, stroke, as well as in people with sleep disorders and the elderly [88,90,93,94,95,96,97,98]. In a study by Da Mesquita et al. [90] significant deposition of amyloid β with macrophage infiltration in the meninges was observed in transgenic mice with Alzheimer’s disease that had damaged meningeal lymphatic vessels. In mice without damage to the meningeal lymphatic vessels, this was not observed. The authors emphasized that similar deposits of amyloid β are found in patients with Alzheimer’s disease, which may accelerate the onset of cognitive deficits associated with this disease [90]. Li et al. [99] showed that infection with neurotropic viruses in mice promoted the expansion of meningeal lymphatic vessels but, at the same time, impairs the removal of macromolecules from the cerebrospinal fluid. Surgical ligation or photodynamic ablation of meningeal lymphatic vessels increased neurological damage and mortality in mice, as these vessels constitute a critical pathway for viral drainage from the CNS to the cervical lymph nodes. Initial treatment with vascular endothelial growth factor C promoted the expansion of functional lymphatic vessels and attenuated the effects of viral infection in mice [99]. In turn, Chen et al. [94] showed the involvement of the meningeal lymphatic vessels in the removal of extravasated erythrocytes from the cerebrospinal fluid to the cervical lymph nodes after subarachnoid hemorrhage (SAH). This finding suggested a new potential therapy for early SAH as well as other types of hemorrhage such as intracranial [94]. The study of Hu et al. [96] indicated that mice with intracranial gliomas or metastatic melanomas experienced significant alterations in the structure of the dorsal meningeal lymphatic vessels. Moreover, the disruption of dorsal meningeal lymphatic vessels was enough to hinder the drainage of fluid inside the tumor and the spread of brain tumor cells to the deep cervical lymph nodes. In addition, the authors demonstrated that the dorsal meningeal lymphatic vessels play a crucial role in generating a potent immune response against brain tumors. They found that the impairment of these vessels significantly decreased the effectiveness of an antitumor combination therapy involving anti-programmed cell death-1 (anti-PD-1) and anti-cytotoxic-T-lymphocyte-associated antigen 4 (CTLA-4) [96]. Bolte et al. [100] observed impaired lymph outflow through the lymphatic vessels of the meninges as a result of traumatic brain injury. These disturbances appeared a few hours after the injury and persisted for at least a month. The authors suggest that increased intracranial pressure as a result of trauma may contribute to meningeal lymphatic dysfunction [100]. Ding et al. [101], in patients with idiopathic Parkinson’s disease, using imaging studies, showed a significantly reduced lymph flow through the meningeal lymphatic vessels along the superior sagittal and sigmoid sinus as well as a noticeable delay in perfusion of deep cervical lymph nodes compared to patients with atypical parkinsonism. The authors also showed that injection of preformed fibrils of α-synuclein led to the development of α-synuclein pathology, which was followed by delayed drainage of meningeal lymphatic vessels, loss of tight junctions between meningeal lymphatic endothelial cells, and increased inflammation of the meninges. Blocking the flow through meningeal lymphatic vessels in treated mice increased α-synuclein pathology and worsened motor and memory deficits, leading to disease progression [101].

To summarize, impaired meningeal lymphatic vessel drainage can contribute to the accumulation of macromolecules, such as amyloid β and α-synuclein in the brain, as well as the removal of neurotropic viruses and tumor cells into cervical lymph nodes. In addition, disrupted meningeal lymphatic vessels can negatively influence the removal of red blood cells from cerebrospinal fluid. Promoting the expansion of functional meningeal lymphatic vessels can be beneficial in treating certain neurological conditions, such as virus infections or brain tumors.

## 11. The Role of Cerebrospinal Fluid Routine Laboratory Examination in the Diagnosis of CNS Diseases

Cerebrospinal fluid laboratory examination is an important element in the diagnosis of CNS diseases, e.g., multiple sclerosis, encephalitis, meningitis, brain tumors, Creutzfeldt–Jakob disease, Alzheimer’s disease, and many others. An increase or decrease in various components of the cerebrospinal fluid, such as concentration of total protein, glucose, neurospecific proteins, and percentage of lymphocytes or neutrophils, may indicate the presence of disease or inflammation. In most cases, analysis of the cerebrospinal fluid allows for the correct diagnosis and implementation of appropriate treatment [70,102]. For example, the detection of oligoclonal bands in the cerebrospinal fluid, which are not present in the serum, is considered the “gold standard” in the diagnosis of multiple sclerosis On the other hand, in patients with Guillain–Barré syndrome, there is an increase in the total protein concentration in the cerebrospinal fluid and the value of Q_Alb_, with normal cell counts [73]. Evaluation of total protein, glucose, lactate, and cytosis (the number of leukocytes per microliter of cerebrospinal fluid) is used in the differential diagnosis of viral and bacterial meningitis [103,104]. If a CNS tumor is suspected, cerebrospinal fluid cytology can help identify atypical cells characteristic of cancers such as CNS lymphoma, leukemia, meningeal metastases, and primary brain tumors [74]. Increased concentration of the Tau protein and decreased concentration of β-amyloid are observed in the cerebrospinal fluid in Alzheimer’s disease [105].

There are many more examples of the use of cerebrospinal fluid analysis in the diagnosis of CNS diseases. Undoubtedly, the examination of cerebrospinal fluid can help identify biomarkers that indicate the presence or severity of the disease and assess the effectiveness of treatment. In addition, it allows us to understand the pathogenesis and disease mechanisms, which is crucial in discovering new strategies of treatment and therapy for patients with CNS diseases. For this reason, research into the search for new biomarkers that will provide more detailed information on CNS diseases and allow for more effective diagnosis and treatment is extremely important.

## 12. Cerebrospinal Fluid Omics-Based Research

Research based on omics, which involves large-scale analysis of biomarkers by molecule types such as genomics, proteomics, and metabolomics, is a new direction in disease diagnosis and treatment [106]. Cerebrospinal fluid is the biological fluid that is in closest proximity to the brain and serves as a direct reflection of the pathological changes occurring in the CNS [107]. Thus, the analysis of cerebrospinal fluid biomarkers using this approach has demonstrated promising potential in the diagnosis and treatment of diseases that affect the CNS [106,108].

Recently, numerous omics research has focused on unraveling the genetic basis of diseases that impact the CNS, such as Alzheimer’s disease, Parkinson’s disease, stroke, multiple sclerosis, and others. This has included genome-wide association studies (GWAS) in particular [106,109,110,111,112,113,114]. For example, GWAS studies allow for the identification of the *apolipoprotein E4* (*APOE4*) allele as the most well-established and significant risk factor for Alzheimer’s disease [109]. Other genetic polymorphisms associated with Alzheimer’s disease encompasses *clusterin* (*CLU*), *sortilin-related receptor-1* (*SORL1*), *complement component receptor 1* (*CR1*), *ATP-binding cassette transporter A member 7* (*ABCA7*), *fermitin family member 2* (*FERMT2*), *major histocompatibility complex class II* (*HLA-DRB5*, *HLA-DRB1*), *bridging integrator 1* (*BIN1*), *phosphatidylinositol-binding clathrin assembly molecule* (*PICALM*), and *aquaporin 4* (*AQP4*) [106,109,115,116,117]. Genetic polymorphisms associated with Parkinson’s disease, also identified using GWAS studies, are *SNCA*, *PARK7*, *PRKN*, *RAB29*, *MAPT*, *BST1*, *GAK*, *LRRK2*, and *HLA-DRB5*, among others [110,111,112].

Studies have shown that many genetic variations are associated with diseases such as Alzheimer’s and Parkinson’s. As a result, researchers have proposed a polygenic hazard score that quantifies an individual’s age-specific genetic risk for these diseases by aggregating the risk across multiple genetic variants [118,119]. This score can predict an individual’s genetic susceptibility to a disease with high accuracy, even while excluding the most common genetic variations such as *APOE*. The accuracy of the polygenic hazard score is supported by its correlation with other biomarkers such as cerebrospinal fluid amyloid β and tau protein levels [112,118,120].

The use of liquid chromatography–mass spectrometry (LC-MS) in analyzing the cerebrospinal fluid proteome is a powerful technique that enables the quantification of multiple proteins without bias [121]. Yang et al. [108] have generated a genomic atlas of protein levels in the brain, cerebrospinal fluid, and plasma, identifying hundreds of protein quantitative trait loci (pQTLs) for each tissue. Using Mendelian randomization, they have nominated proteins implicated in neurological diseases such as Alzheimer’s, Parkinson’s, and stroke [108]. Eninger et al. [122] conducted a study using an advanced proteomics technique to identify changes in over 600 proteins in only 2 µL of murine cerebrospinal fluid. They found that more than 20 glial-derived proteins showed an increase in the cerebrospinal fluid of aged mice with transgenic expression of β-amyloid precursor protein and α-synuclein, which could potentially be used to differentiate and monitor neuroinflammatory disease stages in humans [122]. Bader et al. [123] identified over 1000 proteins that register differing levels between Alzheimer’s disease and non-Alzheimer’s disease cerebrospinal fluid, including well-known neurodegeneration-related proteins such as tau, superoxide dismutase 1 (SOD1), and Parkinson disease protein 7 (PARK7). They also identified a 40-protein signature that consistently shows differential expression in Alzheimer’s disease cerebrospinal fluid. Meanwhile, Wesenhagen et al. [124] found 42 proteins consistently associated with Alzheimer’s disease across multiple studies, with a small subset potentially being altered in mild cognitive impairment. A scalable and sensitive mass-spectrometry-based proteomics workflow has also been developed to examine cerebrospinal fluid proteome profiling, revealing changes in proteins for Parkinson’s disease patients and enhanced neuroinflammation signatures in individuals with *LRRK2* G2019S mutations, the most common genetic cause of autosomal dominant Parkinson’s disease. By comparing cerebrospinal fluid proteomes with urinary proteome profiles, researchers have discovered more than 1000 common proteins, including lysosomal proteins, which could improve our understanding of Parkinson’s disease pathogenesis [125].

Metabolomics is a recently developed method for analyzing the metabolites present in cerebrospinal fluid. Proper interpretation of the metabolite data obtained from this analysis, as well as understanding the resulting biochemical changes, is crucial for gaining insight into neuroinflammatory mechanisms, identifying biomarkers, predicting disease progression, and developing effective treatment strategies [126]. Yan et al. [126] identified several metabolic pathways, including tryptophan-kynurenine, nitric oxide, neopterin, and sphingolipid-ceramide, which are involved in CNS inflammation. The authors focused on CNS infections such as encephalitis, meningitis, and other infections that affect the brain (e.g., hepatitis C, HIV, and malaria), as well as on research related to multiple sclerosis, neurodegeneration, CNS tumors, and autism [126]. Shao et al. [127] emphasized that metabolic imbalances in the metabolism of polyunsaturated fatty acids (PUFAs), bile acids, steroid hormones, caffeine, and amino acids are significant metabolic processes associated with Parkinson’s disease. Analyses of metabolic pathways related to Alzheimer’s disease indicate the involvement of multiple pathways, including those associated with lipoproteins, hemostasis, and the extracellular matrix [124,128]. In addition to the ones mentioned, there exist several other metabolic pathways that are linked to CNS disorders. These include biogenic amines, amino acids, carbohydrates, lipids, and neurotransmitters [126].

## 13. Artificial Cerebrospinal Fluid

For several decades, researchers have been working to develop an artificial cerebrospinal fluid as a means of reducing the use of clinical liquids such as normal saline and lactated Ringer’s solution in neurosurgery. These fluids can potentially cause brain damage, hence the need for a safer alternative [129,130,131,132]. The need to develop artificial cerebrospinal fluid is also dictated by the increasing number of reports indicating the occurrence of complications (headache, fever, convulsions, inflammatory reaction, changes in the cerebrospinal fluid biochemical and cytological laboratory examination) in patients exposed to prolonged irrigation with these fluids [133,134].

Artificial cerebrospinal fluid is a transparent, cell-free liquid designed to mimic the composition of normal cerebrospinal fluid in terms of its electrolyte balance, glucose concentration, pH, and osmotic pressure. The advantage of artificial cerebrospinal fluid compared to irrigation fluids is primarily due to the pH value, which is similar to the pH of normal cerebrospinal fluid [134,135].

Some units prepare their own artificial cerebrospinal fluid [133,136], but this is difficult due to the unstable chemical properties of the glucose and HCO_3_^−^ [136,137,138]. Such prepared artificial cerebrospinal fluid should be used on the day of preparation, and the fluid should be continuously foamed to stabilize the pH [137,138]. Preparing your own artificial cerebrospinal fluid is, therefore, time-consuming and requires specialized equipment and a sterile environment. Ready-to-use artificial cerebrospinal fluid products that do not contain glucose are also available. They should be stored at 4 °C. However, the stability of the formulation is still difficult to maintain as significant changes in pH have been found within a short period of time after opening the package and storing it at room temperature [132]. In recent years, an artificial cerebrospinal fluid known as Artcereb (Artcereb, Otsuka Pharmaceutical Factory, Inc., Tokushima, Japan) has been developed and marketed, which is used as an irrigation and perfusion solution in neurosurgery. Artcereb is characterized by a similar composition and properties to normal human cerebrospinal fluid in terms of glucose and electrolyte concentrations and pH (7.3) [136]. Artcereb is packed in two-chamber bags: one chamber contains electrolytes, and the other contains glucose. The contents of both chambers are mixed under aseptic conditions immediately before use [136].

There are studies in the literature confirming that the use of artificial cerebrospinal fluid leads to less edema and less cytotoxicity in the parenchyma surrounding the rinsed surfaces [136,139] and reduces the permeability of cerebral blood vessels and cell damage [140] compared to the use of normal saline or lactated Ringer’s solution for irrigation. In a rat model of cortical brain injury, artificial cerebrospinal fluid relieves postoperative brain swelling and cell damage [140]. Research by Miyajima et al. [130] showed that human astrocytes cultured in normal saline or lactated Ringer’s solution were characterized by a slight increase in the expression of genes associated with apoptosis and inflammatory response compared to astrocytes cultured in artificial cerebrospinal fluid. Mori et al. [141] indicated that it is particularly important to use artificial cerebrospinal fluid irrigation with an appropriate concentration of Mg^2+^ ions to prevent delayed vasoconstriction in patients with subarachnoid hemorrhage. Artificial cerebrospinal fluid modified with nanoparticles turned out to be useful in protecting the spinal cord against ischemia-reperfusion injury in the rat model [129]. Some reports suggest the need to enrich the artificial cerebrospinal fluid not only with the main divalent cations, such as Ca^2+^ and Mg^2+^, which play a key role in synaptic neurotransmission, but also with a small nanomolar concentration of Zn^2+^ ions mediating in the prevention of cognitive impairment [142,143]. In the available literature, there are also single studies by Shimizu et al. [144] indicating that the use of saline or artificial cerebrospinal fluid (Artcereb) showed no significant effect on cerebral blood flow. However, the authors acknowledge that the use of Artcereb may improve postoperative clinical conditions [144].

## 14. Conclusions

Cerebrospinal fluid is vital for protecting the CNS by providing support, absorbing shocks, and transporting nutrients and wastes. It plays a crucial role in diagnosing neurological conditions and delivering medication directly to the CNS through intrathecal drug administration. Cerebrospinal fluid examination helps identify biomarkers indicating disease presence or severity, evaluates treatment effectiveness, and enables understanding of pathogenesis and disease mechanisms. Therefore, research centered on searching for new biomarkers to provide more detailed information about CNS diseases is necessary to improve diagnosis and discover new treatment methods.

## Figures and Tables

**Table 1:** Comparison of components between cerebrospinal fluid and plasma.

Component	Plasma	Cerebrospinal Fluid
Na^+^ (mmol/L)	153	135–150
K^+^ (mmol/L)	4.7	2.6–3.0
Ca^2+^ (mmol/L)	1.3	1.0–1.4
Mg^2+^ (mmol/L)	0.6	1.2–1.5
Cl^−^ (mmol/L)	110	115–130
Protein (g/L)	60–80	0.15–0.45
Glucose (mmol/L)	3.9–5.5	2.8–4.4
pH	7.4	7.3
Osmolality (mOsm/kg H_2_O)	290	290

Legend for Table 1: Ca^2+^—calcium ions; Cl^−^—chloride ions; H^+^—hydrogen ions; H_2_O—hydrogen monoxide, water; K^+^—potassium ions; Mg^2+^—magnesium ions; Na^+^—sodium ions; pH = −log[H^+^].

**Table 2:** The storage conditions for cerebrospinal fluid, depending on the tests being performed.

Sample Number	Tests	Temperature
1	Chemical and immunological	Freezing the supernatant (−15–−30 °C)
2	Microbiological	Room temperature (25 °C)
3	Cytosis and cytological examination	Cooling down (2–8 °C)

Legend for Table 2: °C—degrees Celsius.

## Data Availability

Not applicable.
